# Serum levels of sLOX‐1 and Lp‐PLA2 can predict the prognosis of acute cerebral infarction with a high specificity

**DOI:** 10.14814/phy2.15160

**Published:** 2022-01-10

**Authors:** Ping Yan, Jing Cao, Yajun Zhou, Xia Zhou, Zhongwu Sun, Xiaoqun Zhu

**Affiliations:** ^1^ Department of Neurology The First Affiliated Hospital of Anhui Medical University Hefei China

**Keywords:** acute cerebral infarction, lipoprotein‐associated phospholipase A2, prognosis, soluble lectin‐like oxidized low‐density lipoprotein receptor‐1

## Abstract

Soluble lectin‐like oxidized low‐density lipoprotein receptor‐1 (sLOX‐1) and lipoprotein‐associated phospholipase A2 (Lp‐PLA2) plays an important role in acute cerebral infarction (ACI), whereas its clinical value in predicting the prognosis is unclear. Thus, this study aimed to explore this issue. A total of 127 ACI patients were included in this prospective observational study. The concentrations of sLOX‐1 and Lp‐PLA2 in serum were measured and their relationship with a poor prognosis 90 days after the onset of ACI was analyzed. We found that patients with poor prognosis had higher mean serum levels of sLOX‐1 and Lp‐PLA2. The level of sLOX‐1 and Lp‐PLA2 could predict the functional outcome of ACI. At the optimal cut off value of sLOX‐1 level (1257.92 ng/ml), the sensitivity and specificity for the poor functional outcome were 0.69 and 0.753, respectively, and the area under ROC curve (AUC) was 0.727. Similarly, the optimal value for Lp‐PLA2 level was 160.9 ng/ml, at which the sensitivity and specificity were 0.643 and 0.835, respectively; and the AUC was 0.758. When the two biomarkers were used in combination, the AUC was 0.855, and the sensitivity and specificity were 0.643 and 0.976, respectively, indicating a significant improvement of the diagnostic specificity. The level of sLOX‐1 or Lp‐PLA2 could thus serve as useful biomarkers to predict the functional outcome of ACI. Combined use of both indicators is better than the use of either single indicator, and provides the highest specificity in predicting poor prognosis.

## Graphical abstract

Our study demonstrates that sLOX‐1 and Lp‐PLA2 serum levels were significantly higher in patients with poor prognosis than those in patients with good prognosis. The level of sLOX‐1 or Lp‐PLA2 could thus serve as useful biomarkers to predict the functional outcome of ACI. Furthermore, the combined use of both indicators is better than the use of either single indicator, and provides the highest specificity in predicting poor prognosis.

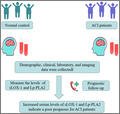

## 1 INTRODUCTION

Stroke has a high incidence, disability, mortality, and recurrence rate, causing a substantial economic and social burden (Wang et al., 2017). Acute cerebral infarction (ACI) is the major subtype of strokes, caused by a sudden interruption in the supply of cerebrovascular blood flow (Allen & Bayraktutan, 2009). Interventional measures such as early identification of ACI, assessment of patient's condition, risk stratification and prognosis assessment, and personalized treatment plan have significant clinical and social significance. At the moment, ACI is usually assessed and diagnosed based on clinical symptoms and radiographic assessment. If the patient has severe clinical symptoms and imaging results show extensive lesions, it may indicate a poor prognosis. Multiple studies have indicated that inflammatory response and various inflammatory factors play an important role in the development of ACI. Furthermore, growing evidence has indicated value of certain laboratory parameters for the management of ACI. However, the sensitivity and specificity of these assays are still relatively low. Therefore, the search for better blood biomarkers for assessing ACI has strong clinical significance.

LOX‐1 (lectin‐like oxidized low‐density lipoprotein receptor‐1) is a scavenger receptor mainly expressed in arterial endothelial cells (Kataoka et al., 1999). It can specifically bind to oxidized low‐density lipoprotein (ox‐LDL) and participate in its oxidation and degradation. Previous studies have shown that LOX‐1 plays an important role in atherosclerosis (Xu et al., 2013) and increases in hypertension, myocardial infarction, and ACI (Hu et al., 2008; Lee et al., 2020; Pothineni et al., 2017; Skarpengland et al., 2018). sLOX‐1 is a proteolytic form of LOX‐1, and studies have shown that sLOX‐1 concentration in plasma can be a reflection of LOX‐1 expression (Murase et al., 2000). Previous studies have shown that sLOX‐1 can be a sensitive marker for the diagnosis of arteriosclerosis‐related diseases (Pirillo & Catapano, 2013; Xu et al., 2013).

Lp‐PLA2, also known as platelet‐activating factor acetyl hydrolase, is an emerging inflammatory marker, mainly released from atherosclerotic plaques by inflammatory cells such as mature macrophages and neutrophils (Asano et al., 1999). Lp‐PLA2 specifically hydrolyzes oxidized phosphatidylcholine from ox‐LDL to produce oxidized free fatty acids and lysophosphatidylcholine, both have atherogenic effects, such as the production of cytokines and adhesion factors (Zalewski & Macphee, 2005), which then promote endothelial dysfunction and the formation and instability of plaques, and ultimately lead to atherosclerotic diseases. Previous studies have shown that elevated levels of Lp‐PLA2 are closely related to ACI (Caslake & Packard, 2005; Gonçalves et al., 2012).

The aim of this study was to further evaluate the diagnostic value of sLOX‐1 and Lp‐PLA2 level in the serum, particularly when used in combination, for predicting the long‐term functional outcome in ACI patients. Our results indicate that the combined use of these two parameters provides the highest specificity in predicting the long‐term functional outcome of ACI.

## 2 MATERIALS AND METHODS

### 2.1 Ethics review

The study was conducted in accordance with the Declaration of Helsinki (as revised in 2013). The study was approved by the Ethics Committee of The First Affiliated Hospital of Anhui Medical University and informed consent was obtained from all individual participants.

### 2.2 Patients

**FIGURE 1:**
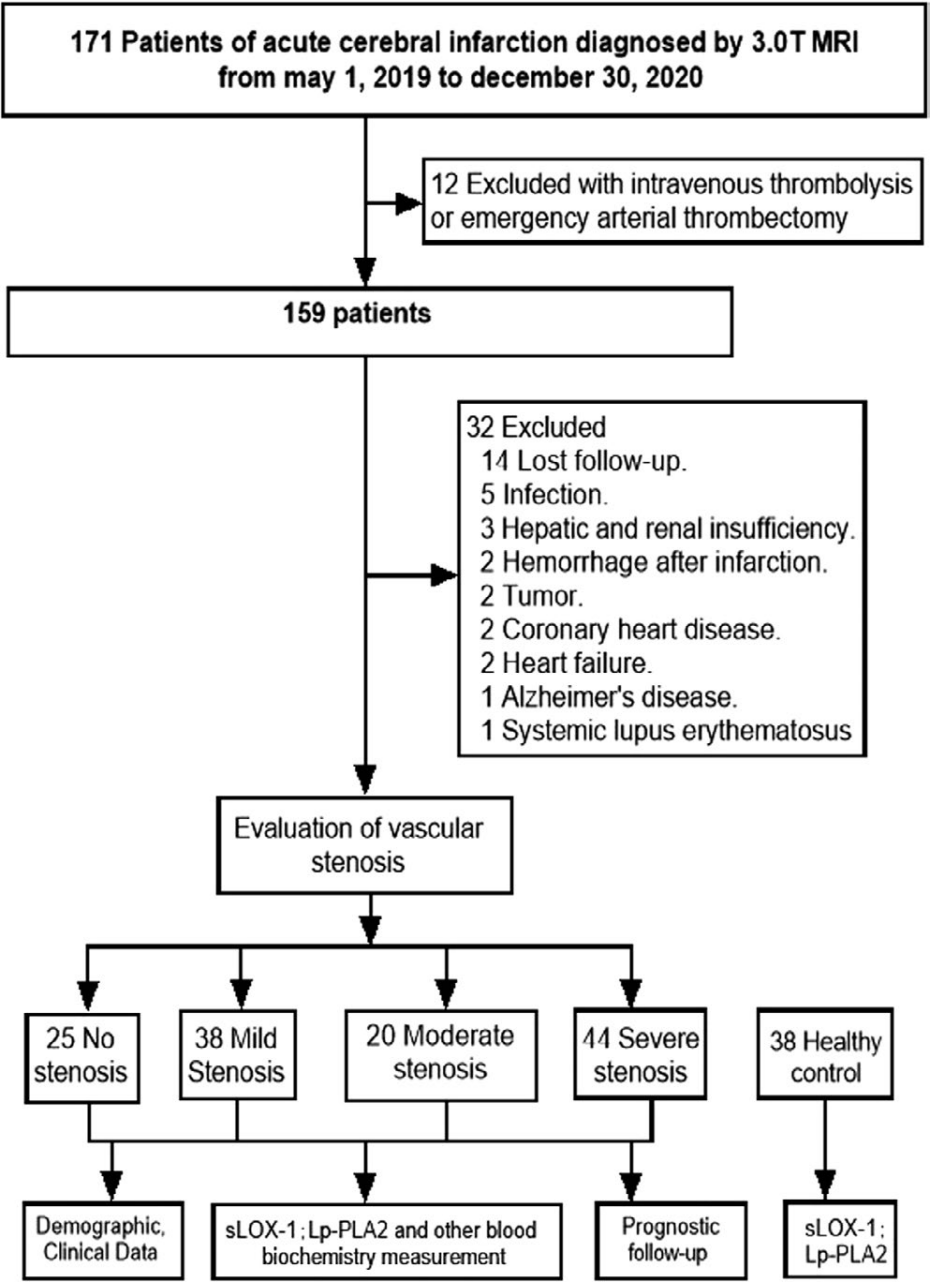
Study flowchart for participant selection. A total of 127 ACI patients with different vascular stenosis were selected based on the screening criteria in this prospective observational study. The concentrations of sLOX‐1 and Lp‐PLA2 in serum were measured and their relationship with a poor prognosis 90 days after the onset of ACI was analyzed

In this study 127 ACI patients (78 males and 49 females) admitted to the First Affiliated Hospital of Anhui Medical University between January 2019 and December 2020 were recruited. All patients were diagnosed with the first episode of ACI within 72 h of the onset of symptoms, which were confirmed by computed tomography (CT) or magnetic resonance imaging (MRI) scans. Patients with the following conditions were excluded from the study: previous history of cerebral infarction, intravenous thrombolysis, or thrombus removal after the onset, any other disease of the central nervous system, such as intracranial hemorrhage, Parkinson's disease, or Alzheimer's disease, serious heart disease, with lung or other infection, cancer, autoimmune system disease, or severe liver and kidney dysfunction. In the control group, 38 healthy subjects were recruited by the physical examination center, including 15 males and 23 females. Complications of cerebrovascular disease, cervical vascular stenosis and autoimmune diseases were excluded. The specific process is shown in Figure 1.

The following information was collected from all participants: age, sex, time from onset to admission, diastolic blood pressure (DBP), systolic blood pressure (SBP), body mass index (BMI), lipid profiles, blood glucose level, family history, smoking, and drinking habits. The severity of ACI was measured using the National Institute of healthy Stroke Scale (NIHSS). The functional recovery at 3 months after onset was assessed by telephone interview using the modified Rankin scale (MRS; Swieten et al., 1988). A poor functional prognosis was defined as MRS score ≥ 3 as previously studied (Kim et al., 2012).

### 2.3 Image analysis

All ACI patients received MRI examination to confirm, CT angiography (CTA) or Magnetic resonance angiography (MRA) were performed to evaluate vascular stenosis. According to the North American Criteria for Symptomatic Carotid Endarterectomy Trial (NASCET, North American Symptomatic Carotid Endarterectomy Trial, 1991), the severity of intracranial/external artery stenosis is divided into mild, moderate, and severe grades.

### 2.4 Blood collection and laboratory test

Blood samples (3 ml) of all subjects were collected within 24 h after admission. The serum was prepared by centrifuging the uncoagulated blood at 3500 r/min for 8 min and allocated before being stored at −80℃ for analysis. The serum sLOX‐1 and Lp‐PLA2 levels were measured by enzyme‐linked immunosorbent assay (ELISA). Other blood chemistry parameters, were determined in the hospital's laboratory department, which include high‐sensitivity C‐reactive protein (hs‐CRP), fasting blood glucose (FBG), total cholesterol (TC), triacylglycerol (TG), creatinine (CRE), urea nitrogen (UN), uric acid (UA), high density lipoprotein cholesterol (HDL‐C), low‐ density lipoprotein cholesterol (LDL‐C), and other blood lipid indicators.

### 2.5 Statistical analyses

The data were analyzed using the software program SPSS Statistics (version 25). Categorical variables were presented as count (%) and compared using the Chi‐square test. Continuous variables of normal distribution and approximately normal distribution are represented by the mean value ± standard deviation, continuous variables of non‐normal distribution are represented by medians with interquartile ranges (25th–75th percentiles). *t*‐test was used for comparison between two groups conforming to normal distribution, and ANOVA analysis was used for comparison between multiple groups. The Mann–Whitney *U* test is used to compare non‐normally distributed continuous variables. Multivariate logistic regression analysis was used to determine the risk factors affecting the prognosis of ACI. Finally, by constructing ROC curve to evaluate the prognostic value of sLOX‐1 and Lp‐PLA2 in predicting ACI prognosis. *p* < 0.05 in a two‐tailed test was considered statistically significant.

## 3 RESULTS

### 3.1 Baseline characteristics

In the present study, a total of 165 individuals were studied, which include the healthy control group (*n* = 38), ACI with no stenosis group (*n* = 25), ACI with mild stenosis group (*n* = 38), ACI with moderate stenosis group (*n* = 20), and ACI with severe stenosis group (*n* = 44) based on the history of ACI and the degree of cerebral vascular stenosis. The demographic and history information are shown in Table 1. The serum levels of sLOX‐1, Lp‐PLA2, and other biochemical elements in these groups were then compared (Table 2). ANOVA analysis showed that sLOX‐1 and Lp‐PLA2 were significantly different among groups (sLOX‐1, *F* = 3.18, *p* = 0.015; Lp‐PLA2, *F* = 2.122, *p* = 0.008). In addition, there were statistically significant differences in the levels of TC, HDL‐C, and UA.

**TABLE 1:** The demographic and history information of ACI patients and healthy controls

Variable	Healthy control (*n* = 38)	No stenosis (*n* = 25)	Mild stenosis (*n* = 38)	Moderate stenosis (*n* = 20)	Severe stenosis (*n* = 44)	Statistics	*p* value
Age (years)	58.42 ± 8.93	75.4 ± 11.72	62.08 ± 11.25	61.4 ± 16.04	66.75 ± 13.53	8.464^a^	<0.001
Male, *N* (%)	15 (39.4)	13 (52.0)	25 (65.8)	12 (60.0)	28 (63.6)	7.028^b^	0.134
BMI (kg/m^2^)	24.51 ± 3.05	24.22 ± 3.67	24.27 ± 3.32	24.07 ± 3.6	23.84 ± 2.82	0.206^a^	0.935
Hypertension, *N* (%)	8 (21.0)	14 (56.0)	26 (68.4)	6 (30.0)	18 (40.9)	24.722^b^	<0.001
SBP (mmHg)	138.67 ± 19.47	153.52 ± 23.03	151.53 ± 22.14	146 ± 19.83	152.68 ± 22.02	1.127^a^	0.347
DBP (mmHg)	77.33 ± 9.37	81.68 ± 10.54	85.53 ± 21.79	80.4 ± 10.96	85.95 ± 14.6	1.047^a^	0.386
Diabetes, *N* (%)	1 (2.6)	3 (12.0)	6 (15.8)	4 (20.0)	10 (22.7)	8.941^b^	0.063
Smoking, *N* (%)	5 (13.1)	5 (20.0)	9 (23.7)	7 (0.35)	6 (13.6)	5.448^b^	0.244
Alcohol drinking, *N* (%)	8 (21.0)	3 (12.0)	6 (15.8)	5 (0.25)	1 (2.3)	10.627^b^	0.031

Note

Superscript a indicates the *F* value obtained by ANOVA analysis; superscript b indicates the value of *χ* ^2^ obtained by the chi‐square test.

Abbreviations: BMI, body mass index; DBP, diastolic blood pressure; SBP, systolic blood pressure.

**TABLE 2:** The blood biochemistry measurement of ACI patients and healthy controls

Variable	Healthy control (*n* = 38)	No stenosis (*n* = 25)	Mild stenosis (*n* = 38)	Moderate stenosis (*n* = 20)	Severe stenosis (*n* = 44)	*F* value	*p* value
sLOX‐1 (pg/ml)	1063.41 ± 251.40	1064.53 ± 242.86	1173.89 ± 234.8	1158.07 ± 314.91	1250.99 ± 310.2	3.18	0.015
Lp‐PLA2 (ng/L)	114.24 ± 43.62	133.74 ± 59.35	135.75 ± 54.67	116.42 ± 37.27	144.83 ± 62.39	2.122	0.08
hs‐CRP (mg/L)	1.85 ± 2.36	3.68 ± 4.89	4.38 ± 6.46	1.92 ± 2.39	4.34 ± 6.76	1.927	0.108
TC (mmol/L)	4.93 ± 1.06	4.34 ± 0.85	4.04 ± 1.02	4.23 ± 0.69	4.23 ± 1.32	3.818	0.005
TG (mmol/L)	1.52 ± 1.02	1.34 ± 0.6	1.55 ± 0.73	1.31 ± 0.55	1.59 ± 1.3	0.484	0.748
HDL‐C (mmol/L)	1.45 ± 0.45	1.27 ± 0.41	1.07 ± 0.26	1.25 ± 0.35	1.24 ± 0.47	4.279	0.003
LDL‐C (mmol/L)	2.16 ± 1.10	2.54 ± 0.9	2.39 ± 0.92	2.4 ± 0.77	2.44 ± 0.93	0.306	0.873
Urea (mmol/L)	5.18 ± 1.02	5.77 ± 1.41	8.39 ± 9.35	4.9 ± 1.31	6.32 ± 8.54	0.606	0.659
Cr (umol/L)	66.23 ± 14.94	79.44 ± 31.7	68.95 ± 17.41	65.52 ± 14.5	68.95 ± 16.83	2.154	0.077
UA (umol/L)	320.71 ± 71.68	355.2 ± 100.94	281.71 ± 100.47	302.4 ± 94.35	305.52 ± 84.77	2.73	0.031
WBC (10^9^/L)	7.14 ± 3.06	6.78 ± 2.44	7.26 ± 2.58	7.09 ± 1.95	6.74 ± 1.73	0.351	0.843
NEUT (10^9^/L)	4.93 ± 2.68	4.54 ± 2.17	4.92 ± 2	4.81 ± 1.5	6.17 ± 11.77	0.305	0.874
LYMPH (10^9^/L)	1.72 ± 0.76	1.79 ± 0.51	2.3 ± 3.05	1.77 ± 0.77	2.22 ± 3.56	0.285	0.887
FBG (mmol/L)	6.06 ± 0.98	6.36 ± 2.55	7.74 ± 3.59	7.32 ± 3.5	6.71 ± 2.32	2.284	0.063

Note

Statistics analysis is performed by ANOVA.

Abbreviations: CRE, creatinine; FBG, fasting blood glucose; HDL‐C, high‐density lipoprotein cholesterol; hs‐CRP, high‐sensitivity C‐reactive protein; LDL‐C, low‐density lipoprotein cholesterol; Lp‐PLA2, lipoprotein‐associated phospholipase A2; LYMPH, lymphocyte; NEUT, neutrophil; sLOX‐1, soluble lectin‐like oxidized low‐density lipoprotein receptor‐1; TC, total cholesterol; TG, triacylglycerol; UA, uric acid; UN, urea nitrogen; WBC, leukocyte.

### 3.2 Multivariate logistic regression analyses for outcome

We aimed to investigate the potential relationship of blood levels of sLOX‐1 and Lp‐PLA2 with the long‐term outcomes of ACI. All patients were followed up to 3 months after the onset using MRS to assess the functional status. Among them, 85 patients had a good outcome (MRS Score < 3) and 42 patients had a poor outcome (MRS Score ≥ 3). The characteristics of patients with good and poor outcomes were then examined. The demographic and other history information did not present notable differences between the two groups (Table 3). However, there was a significant difference in the levels of Lp‐PLA2 and sLOX‐1 between these two groups (*p* < 0.05; Table 4). Patients who had poor outcome had higher mean serum sLOX‐1 level (*p* < 0.001) and Lp‐PLA2 level (*p* < 0.001) than who had good outcome.

**TABLE 3:** The demographic and history information of good prognosis and poor prognosis

Variable	Good prognosis (*n* = 85)	Poor prognosis (*n* = 42)	Statistics	*p* value
Age (years)	66.76 ± 13.15	65.1 ± 15.11	−0.64^a^	0.523
Male, *N* (%)	54 (63.5)	24 (57.1)	0.484^b^	0.487
BMI (kg/m^2^)	24.12 ± 3.46	24 ± 2.8	−0.182^a^	0.856
Hypertension, *N* (%)	47 (55.3)	24 (57.1)	0.039^b^	0.843
SBP (mmHg)	151.53 ± 21.97	151.29 ± 21.76	−0.059^a^	0.953
DBP (mmHg)	84.93 ± 16.79	82.45 ± 14.37	−0.819^a^	0.414
Diabetes, *N* (%)	14 (16.5)	10 (23.8)	0.988^b^	0.32
Smoking, *N* (%)	20 (23.5)	5 (11.9)	2.403^b^	0.121
Alcohol drinking, *N* (%)	8 (9.4)	4 (9.5)	0^b^	1
NIHSS	2 (1, 5)	3 (1, 7.25)	−0.660^c^	0.509

Note

Superscript a indicates the *t* value obtained by the *t*‐test; superscript b indicates the value of *χ* ^2^ obtained by the chi‐square test; superscript c indicates the *Z* value obtained by the Mann–Whitney *U* test.

Abbreviations: BMI, body mass index; DBP, diastolic blood pressure; NIHSS, National Institutes of Health Stroke Scale; SBP, systolic blood pressure.

**TABLE 4:** The blood biochemistry measurement of good prognosis and poor prognosis

Variable	Good prognosis (*n* = 85)	Poor prognosis (*n* = 42)	*t* value	*p* value
sLOX‐1 (pg/ml)	1098.83 ± 244.4	1333.95 ± 290.94	4.784	<0.001
Lp‐PLA2 (ng/L)	117.59 ± 42.11	171.62 ± 64.21	4.953	<0.001
hs‐CRP (mg/L)	2.85 ± 5.16	5.84 ± 6.63	2.569	0.012
TC (mmol/L)	4.1 ± 1.09	4.39 ± 0.97	1.467	0.145
TG (mmol/L)	1.42 ± 1.01	1.62 ± 0.74	1.184	0.239
HDL‐C (mmol/L)	1.22 ± 0.41	1.15 ± 0.34	−0.906	0.367
LDL‐C (mmol/L)	2.35 ± 0.86	2.62 ± 0.94	1.592	0.114
Urea (mmol/L)	7.33 ± 14.26	5.15 ± 1.19	−0.987	0.325
Cr (umol/L)	71.69 ± 20.69	68.02 ± 20.87	−0.937	0.351
UA (umol/L)	319.74 ± 104.87	283.29 ± 72.89	−2.279	0.025
WBC (10^9^/L)	7.05 ± 2.2	6.78 ± 2.14	−0.644	0.521
NEUT (10^9^/L)	5.71 ± 8.55	4.36 ± 1.79	−1.012	0.313
LYMPH (10^9^/L)	1.96 ± 2.09	2.34 ± 3.64	0.745	0.458
FBG (mmol/L)	7.29 ± 3.3	6.55 ± 2.24	−1.311	0.192

Note

Statistical analysis is performed by *t*‐test.

Abbreviations: CRE, creatinine; FBG, fasting blood glucose; HDL‐C, high‐density lipoprotein cholesterol; hs‐CRP, high‐sensitivity C‐reactive protein; LDL‐C, low‐density lipoprotein cholesterol; Lp‐PLA2, lipoprotein‐associated phospholipase A2; LYMPH, lymphocyte; NEUT, neutrophil; sLOX‐1, soluble lectin‐like oxidized low‐density lipoprotein receptor‐1; TC, total cholesterol; TG, triacylglycerol; UA, uric acid; UN, urea nitrogen; WBC, leukocyte.

To further examine the different risk factors affecting the prognosis of ACI we performed multivariate logistic analysis. After adjusting all the confounders, we found that sLOX‐1 and Lp‐PLA2 levels were associated with a poor functional outcome (sLOX‐1, odds ratio = 1.005, *p* < 0.001; Lp‐PLA2, odds ratio = 1.025, *p* < 0.001; Table 5). The results confirmed that the serum level of sLOX‐1 and the serum level of Lp‐PLA2 can be independent risk factors for ACI patients with a poor prognosis. The levels of UA and hs‐CRP, while significantly different between the good and poor outcome groups (Table 4), do not seem to be independent risk factors for poor prognosis (Table 5).

**TABLE 5:** Multivariate logistic regression analyses for poor outcome

Variable	*β*	Wald	OR	95% CI	*p* value
sLOX‐1	0.005	16.031	1.005	1.002–1.007	<0.001
Lp‐PLA2	0.024	18.073	1.025	1.013–1.036	<0.001
hs‐CRP	0.024	0.337	1.024	0.945–1.111	0.561
UA	−0.003	1.076	0.997	0.992–1.002	0.300

Abbreviations: 95% CI, 95% confidence interval; hs‐CRP, high‐sensitivity C‐reactive protein; Lp‐PLA2, lipoprotein‐associated phospholipase A2; OR, odds ratio; sLOX‐1, soluble lectin‐like oxidized low‐density lipoprotein receptor‐1; UA, uric acid.

### 3.3 sLOX‐1 and Lp‐PLA2 prediction of prognosis of acute cerebral infarction

**FIGURE 2:**
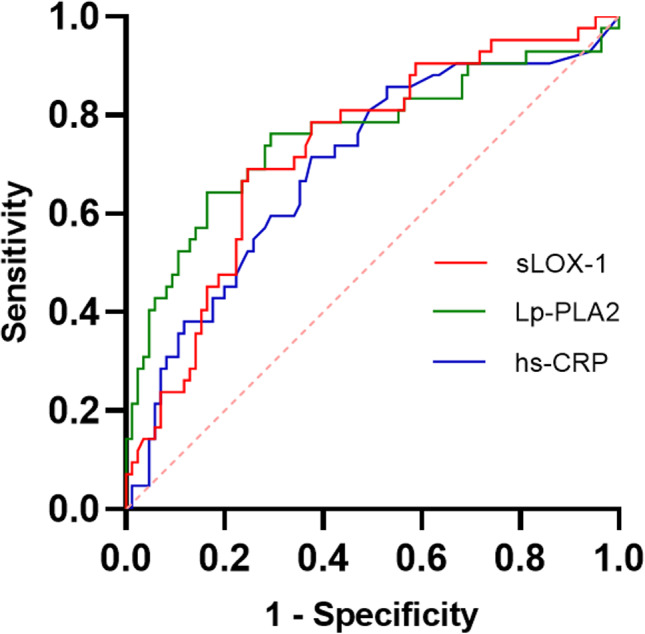
ROC curves of sLOX‐1, Lp‐PLA2, and hs‐CRP for predicting poor prognosis of ACI. The ROC curves of sLOX‐1, Lp‐PLA2, and hs‐CRP for predicting poor prognosis of ACI were constructed. The AUC for sLOX‐1 level is 0.727 (95% confidence interval: 0.634–0.8, *p* < 0.001). The AUC for Lp‐PLA2 level is 0.758 (95% confidence interval: 0.659–0.856, *p* < 0.001). The AUC for hs‐CRP level is 0.694 (95% confidence interval: 0.595–0.794, *p* < 0.001)

These findings suggested that sLOX‐1 and Lp‐PLA2 may serve as potential diagnostic markers for prediction of long‐term functional recovery from ACI. We thus determined the diagnostic value of the serum sLOX‐1 and Lp‐PLA2 levels by constructing the ROC curve (Figure 2). We found that the levels of sLOX‐1 and Lp‐PLA2 could be used to predict the functional outcome of ACI. At the proper cut off levels, the sensitivity and specificity of the two markers are comparable with the AUC of 0.727 and 0.758, respectively. The performance of hs‐CRP was lower with an AUC of 0.694 (Table 6).

**TABLE 6:** Evaluation of the effect of various indexes on prognosis of ACI

Variable	AUC	*p* value	95% CI	Cutoff value	Maximum index	Sensitivity	Specificity
sLOX‐1	0.727	<0.001	0.634–0.82	1257.9151	0.443	0.69	0.753
Lp‐PLA2	0.758	<0.001	0.659–0.856	160.9	0.478	0.643	0.835
hs‐CRP	0.694	<0.001	0.595–0.794	1.65	0.338	0.714	0.624
sLOX‐1 + Lp‐PLA2 + hs‐CRP	0.855	<0.001	0.778–0.933	0.4680541	0.631	0.69	0.941
sLOX‐1 + Lp‐PLA2	0.854	<0.001	0.777–0.932	0.5333486	0.619	0.643	0.976
sLOX‐1 + hs‐CRP	0.767	<0.001	0.68–0.854	0.2378389	0.421	0.833	0.588
Lp‐PLA2 + hs‐CRP	0.77	<0.001	0.672–0.868	0.3925918	0.478	0.643	0.835

Note

sLOX‐1 + Lp‐PLA2 + hs‐CRP, combination of sLOX‐1, Lp‐PLA2, and hs‐CRP; sLOX‐1 + Lp‐PLA2, combination of sLOX‐1 and Lp‐PLA2; sLOX‐1 + hs‐CRP, combination of sLOX‐1 and hs‐CRP; Lp‐PLA2 + hs‐CRP, combination of Lp‐PLA2 and hs‐CRP.

Abbreviations: 95% CI, 95% confidence interval; AUC, area under ROC curve; hs‐CRP, high‐sensitivity C‐reactive protein; Lp‐PLA2, lipoprotein‐associated phospholipase A2; OR, odds ratio; sLOX‐1, soluble lectin‐like oxidized low‐density lipoprotein receptor‐1; UA, uric acid.

**FIGURE 3:**
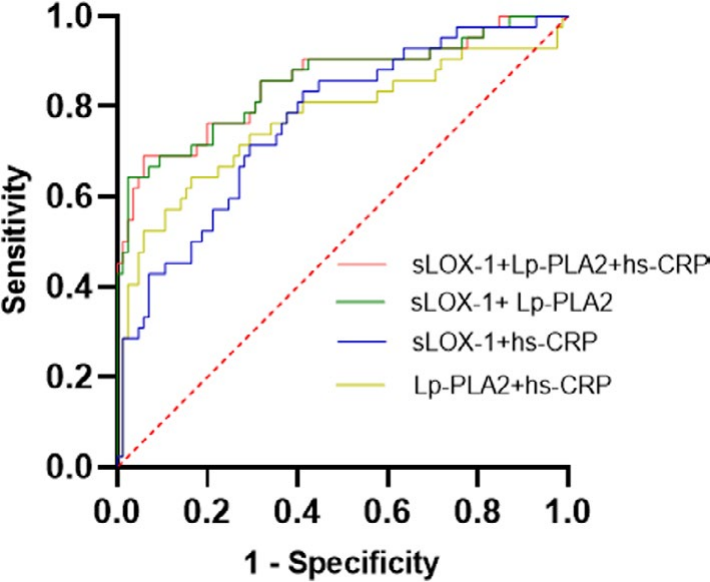
The ROC curves of sLOX‐1, Lp‐PLA2, and hs‐CRP in different combinations for predicting poor prognosis of ACI. The ROC curves of different combinations of sLOX‐1, Lp‐PLA2, and hs‐CRP for predicting poor prognosis of ACI were constructed. The AUC for the combination of sLOX‐1 and Lp‐PLA2 was 0.854 (95% confidence interval: 0.777–0.932, *p* < 0.001). The AUC for the combination of sLOX‐1 and hs‐CRP is 0.767 (95% confidence interval: 0.68–0.854, *p* < 0.001). The AUC for the combination of Lp‐PLA2 and hs‐CRP is 0.77 (95% confidence interval: 0.672–0.868, *p* < 0.001). The AUC for the combination of sLOX‐1, Lp‐PLA2, and hs‐CRP is 0.855 (95% confidence interval: 0.778–0.933, *p* < 0.001)

We then hypothesized that the combined use of these parameters may provide better predicting powers. ROC curves were constructed for the different combinations of sLOX‐1, Lp‐PLA2, and hs‐CRP (Figure 3). When sLOX‐1 and Lp‐PLA2 were used in combination the sensitivity was not improved but the specificity had increased to a much higher level of 0.976 with a higher AUC of 0.854 (Table 6). The combination of hs‐CRP with either sLOX‐1 or Lp‐PLA2 alone or together did not further increase the performance (Figure 3; Table 6). Taken together these results indicate that a combined use of the serum levels of sLOX‐1 and Lp‐PLA2, as a diagnostic marker, may be clinically useful for risk stratification and prediction of long‐term functional recovery from ACI with a high specificity.

## 4 DISCUSSION

Our results indicated that sLOX‐1 and Lp‐PLA2 could serve as independent risk factor for poor prognosis in ACI, and can thus be used as a diagnostic marker for prediction of long‐term functional outcome in ACI. The combination of the two molecular signatures provides the highest specificity of the prediction. To our knowledge, this is the first study of a combined use of sLOX‐1 and Lp‐PLA2 to predict long‐term prognosis of ACI.

LOX‐1 is mainly expressed in arterial endothelial cells (Kataoka et al., 1999). Under physiological conditions, the expression of LOX‐1 is very low, but in some disease states, its expression is increased through the action of oxLDL, pro‐inflammatory cytokines, and reactive oxygen (Hofmann et al., 2017; Kataoka et al., 1999). sLOX‐1, a proteolytically cleaved form of LOX‐1, goes into circulation after being liberated from the plasma membrane (Murase et al., 2000). The concentration of sLOX‐1 in plasma is related to the level of receptor expression (Brinkley et al., 2008), and it was suggested that high levels of sLOX‐1 could be a biomarker for vulnerability to atherosclerotic plaques (Hayashida et al., 2005). Multiple studies have indicated that sLOX‐1 plays an important role in the development of ACI (Li et al., 2018; Skarpengland et al., 2018; Yokota et al., 2016). Our study shows that elevated sLOX‐1 is an independent risk factor for poor prognosis in ACI and can be used to predict long‐term functional outcome of ACI, which may be related to the role of LOX‐1 in causing endothelial dysfunction, plaque initiation, progression, and instability (Huang et al., 2017; Li et al., 2018).

Lp‐PLA2 is a newly defined vascular‐specific inflammatory factor whose level is elevated in response to vascular damage with a high specificity (Thompson et al., 2010), and is thus considered as a potential biomarker of vascular inflammation and plaque formation (Yang et al., 2017). Previous studies have demonstrated that Lp‐PLA2 is closely associated with arteriosclerosis‐related diseases such as coronary atherosclerosis, cerebrovascular stenosis, TIA, and ACI. A number of studies suggested that elevated serum Lp‐PLA2 are more closely associated with large vessel stenosis and can be used as a potential biomarker for predicting cerebrovascular stenosis levels in ACI patients (Cao et al., 2021; Gonçalves et al., 2012; Hu et al., 2019; Oei et al., 2005). However, our study goes one step further by indicating that elevated Lp‐PLA2 level is associated with poor prognosis of ACI and is an independent risk factor for *poor prognosis* of ACI, suggesting that elevated serum Lp‐PLA2 level can serve as an early indicator for long‐term prognosis prediction. This conclusion is also supported by previous studies showing Lp‐PLA2 level being correlated with the admission severity in ACI patients (Lin et al., 2015; Zhou et al., 2018), and with the recurrence of ACI and TIA (Han et al., 2017; Lin et al., 2015).

Based on the ROC curves, we found that serum sLOX‐1 and Lp‐PLA2 levels have high diagnostic values for predicting long‐term functional outcomes after ACI, and the combination of the serum sLOX‐1 and Lp‐PLA2 can significantly improve the specificity of prediction without scarifying the sensitivity. Serum level of hs‐CRP is another biomarker that has been studied for ACI (Wang et al., 2021). However, combining hs‐CRP with sLOX‐1 improved the sensitivity at the expense of specificity, whereas combining hs‐CRP with Lp‐PLA2 did not yield any improvement. Finally, inclusion of hs‐CRP in the combination with sLOX‐1 and Lp‐PLA2 actually decreased the performance of the assay. We thus conclude that sLOX‐1 and Lp‐PLA2 offer the best combination with highest specificity to predicate a poor prognosis with sufficient sensitivity.

The use of serum sLOX‐1 and Lp‐PLA2 levels to predict the functional outcome of ACI will be beneficial to the risk stratification, prognosis assessment, and individualized treatment. Both sLOX‐1 and Lp‐PLA2 have also been studied to be therapeutic targets for atherosclerosis‐related diseases. At present, some lipid‐lowering agents (Agouridis et al., 2011; Albert et al., 2005) and selective Lp‐PLA2 inhibitor Darapladib (Serruys et al., 2008) have been shown to reduce Lp‐PLA2 activity level, but there is no concrete evidence to suggest that such treatment can reduce the incidence and mortality of cerebrovascular events. Clearly, more studies are needed in the future to determine whether sLOX‐1 and Lp‐PLA2 can be used as a therapeutic target for cerebrovascular disease.

We recognize that the present study has its limitations. First, our study was conducted in one hospital and had a small sample size. Second, our study did not consider the dynamic changes of sLOX‐1 and Lp‐PLA2 after the onset of cerebral infarction. Therefore, it is necessary to further study the dynamic changes of sLOX‐1 and Lp‐PLA2. Third, regular medication and rehabilitation are still a key part of functional recovery after ACI. However, our study did not establish the rehabilitation training and medication status of patients after discharge from the hospital. Further studies on these issues are necessary. Our findings would thus support the rationale to conduct large scale multicenter prospective studies to further confirm the utility of the assay in larger and more diverse ACI patient populations.

## 5 CONCLUSION

In conclusion, the present study suggested that sLOX‐1 and Lp‐PLA2 levels were increased significantly in ACI patients with poor prognosis compared to those with good prognosis and the two biomarkers were considered as risk factors for poor prognosis. The level of sLOX‐1 or Lp‐PLA2 could be a potential biomarker to predict the functional outcome of ACI. A much more specific result can be obtained by combining these two indicators.

## CONFLICT OF INTEREST

The authors declare that they have no conflict of interest.

## AUTHOR CONTRIBUTIONS

Ping Yan: design and perform experiments, identification of cases, analyze data, and wrote the manuscript. Jing Cao, Yajun Zhou and Xia Zhou: identification of cases and editing the manuscript. Zhongwu Sun: develop concept, provide discussion, and identification of cases. Xiaoqun Zhu: conceptualization, experimental design, and wrote the manuscript.
